# Effect of genotype and age on cerebral [^18^F]FDG uptake varies between transgenic APP_Swe_-PS1_dE9_ and Tg2576 mouse models of Alzheimer’s disease

**DOI:** 10.1038/s41598-019-42074-4

**Published:** 2019-04-05

**Authors:** Anniina Snellman, Jatta S. Takkinen, Francisco R. López-Picón, Olli Eskola, Olof Solin, Juha O. Rinne, Merja Haaparanta-Solin

**Affiliations:** 1 MediCity Research Laboratory, University of Turku, Tykistökatu 6 A, FI-20520 Turku, Finland; 2 PET Preclinical Laboratory, Turku PET Centre, University of Turku, Tykistökatu 6 A, FI-20520 Turku, Finland; 3 Doctoral Programme in Clinical Research, University of Turku, Turku, Finland; 4 Radiopharmaceutical Chemistry Laboratory, Turku PET Centre, University of Turku, Kiinamyllynkatu 4-8, FI-20520 Turku, Finland; 5 Accelerator Laboratory, Turku PET Centre, Åbo Akademi University, Kiinamyllynkatu 4-8, FI-20520 Turku, Finland; 6 Department of Chemistry, University of Turku, Vatselankatu 2, FI-20500 Turku, Finland; 7 Turku PET Centre, Turku University Hospital, Kiinamyllynkatu 4-8, FI-20520 Turku, Finland; 8 Division of Clinical Neurosciences, Turku University Hospital, Kiinamyllynkatu 4-8, FI-20520 Turku, Finland

## Abstract

Back-translation of clinical imaging biomarkers of Alzheimer’s disease (AD), such as alterations in cerebral glucose metabolism detected by [^18^F]FDG positron emission tomography (PET), would be valuable for preclinical studies evaluating new disease-modifying drugs for AD. However, previous confounding results have been difficult to interpret due to differences in mouse models and imaging protocols between studies. We used an equivalent study design and [^18^F]FDG µPET imaging protocol to compare changes in cerebral glucose metabolism in commercial transgenic APP_Swe_-PS1_dE9_ (*n* = 12), Tg2576 (*n* = 15), and wild-type mice (*n* = 15 and 9). Dynamic [^18^F]FDG scans were performed in young (6 months) and aged (12 or 17 months) mice and the results verified by *ex vivo* methods (i.e., tissue counting, digital autoradiography, and beta-amyloid and Iba-1 immunohistochemistry). [^18^F]FDG uptake exhibited significant regional differences between genotypes (TG < WT) and ages (6 months <12 months) in the APP_Swe_-PS1_dE9_ model, whereas similar differences were not present in Tg2576 mice. In both models, only weak correlations were detected between regional beta-amyloid deposition or microgliosis and [^18^F]FDG uptake. By using equivalent methodology, this study demonstrated differences in cerebral glucose metabolism dysfunction detected with [^18^F]FDG PET between two widely used commercial AD mouse models.

## Introduction

In recent years, there has been great interest in back-translating clinical molecular imaging methods and biomarkers to preclinical Alzheimer’s disease (AD) research to enable longitudinal follow-up of various pathological changes in transgenic (TG) mouse models and increase the quality of preclinical studies evaluating novel disease-modifying drugs for AD. One such method is positron emission tomography (PET) combined with a glucose analogue 2-deoxy-2-[^18^F]fluoro-D-glucose ([^18^F]FDG) to visualize cerebral glucose metabolism, which reflects regional synaptic and neuronal activity. Regional hypometabolic patterns visualized by [^18^F]FDG PET are an established feature of AD^1^. In addition, regional hypometabolism can predict cognitive decline and conversion to AD in persons with mild cognitive impairment^2,3^ and distinguish AD from other forms of dementia and brain diseases^4^. Studies using TG mouse models of AD have also shown that metabolic dysfunction is associated with neuroinflammation, and that both are early pathological features of AD, emerging before beta-amyloid (Aβ) deposition^5–7^.

In theory, small animal PET (μPET) imaging provides a translational tool for following metabolic changes in different TG mouse models and monitoring the properties and efficacy of novel disease-modifying or symptomatic drugs. However, cerebral glucose hypometabolism has been shown to be less evident in AD mouse models than in AD patients. Depending on the group, study design, animal age, and AD mouse model, increased^8–12^, decreased^6,13–17^, or unchanged^18^ cerebral glucose metabolism has been demonstrated compared to age-matched wild-type (WT) control animals. These inconsistent results have been speculated to have arisen mainly from the heterogenic phenotypes of the selected TG mouse models and methodological differences between laboratories.

The aim of the present study was to use two different TG mouse models of AD, APP_Swe_-PS1_dE9_ and Tg2576 mice, and identical study designs and experimental protocols to compare their suitability for future interventional imaging studies. Both of the chosen models are widely used, commercially available, and present pathological changes typical to AD, such as extracellular Aβ deposition and related neuroinflammation as they age, but at different rate^19–21^. Both models were imaged at 6 months, but due to this known difference in the rate of pathological changes between the models, the second chosen time point to present abundant amyloidosis was 12 months for APP_Swe_-PS1_dE9,_ and 17 months for Tg2576. By using the same experimental protocols, imaging methodology, and analytical methods, we wanted to gain comparable information on the usability of these models in future studies.

## Results

### Experimental design

**Figure 1:**
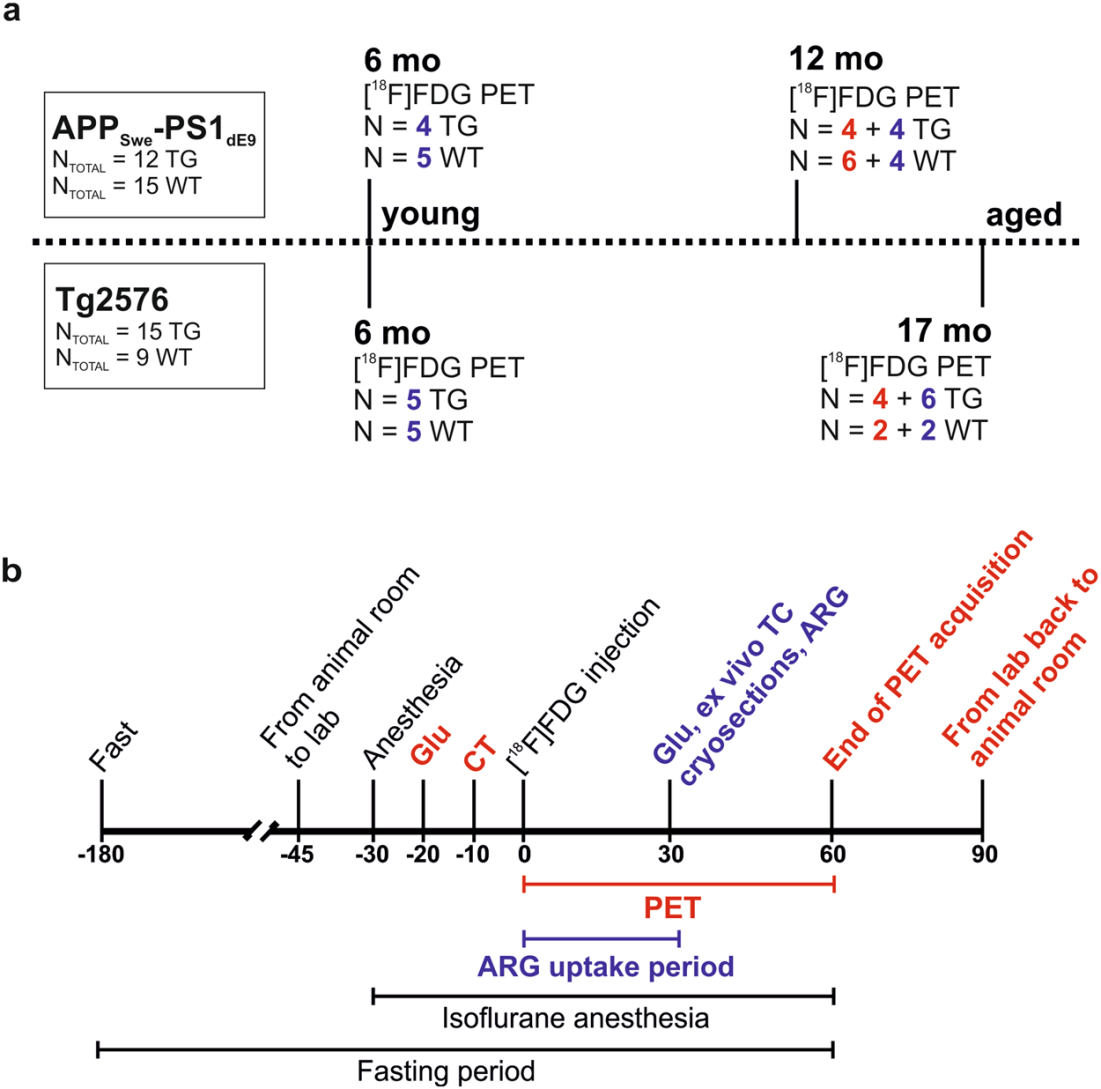
Study design. (**a**) Timeline and number of transgenic (TG) and wild-type (WT) animals used for each time point of the cross-sectional study. Two batches of animals were used due to availability. First batch is indicated with red color and second batch with blue color. (**b**) Used study protocols for *in vivo* imaging (indicated by black and red colors) and *ex vivo* tissue counting (TC) and autoradiography (indicated by black and blue colors). ARG = autoradiography; CT = computed tomography; Glu = Individual blood glucose level measurement.

The experimental study design is presented in Fig. 1, and characteristics of the evaluated groups in Table 1.

**Table 1:** Descriptive statistics for the experimental animals.

Strain	APP_Swe_-PS1_dE9_				Tg2576			
Age (months)	6		12		6		17	
Genotype	TG	WT	TG	WT	TG	WT	TG	WT
**n**	4	5	8	10	5	5	10	4
**n dropouts**	0	1	0	0	0	0	1	0
**PET imaging (n)**	4	5	8	10	5	5	10	4
*Injected dose (MBq)*	6.3 (0.2)	6.2 (0.8)	7.2 (1.3)	6.5 (0.9)	5.5 (1.0)	5.8 (0.3)	6.4 (1.2)	6.3 (0.4)
*Weight (g)*	22.4 (1.3)	26.0 (2.0)	29.2 (4.7)	29.0 (4.5)	25.7 (3.3)	24.3 (2.2)	24.6 (1.9)	30.6 (7.8)
*Glucose (mmol/l)*	10.4 (2.4)	11.3 (0.4)	10.5 (2.5)	10.6 (0.9)	10.0 (2.1)	9.8 (2.5)	7.9 (1.9)	8.1 (1.1)
*Temperature (*°*C)*	36.2 (1.2)	37.4 (0.2)	37.1 (0.8)	37.6 (0.4)	36.5 (1.1)	37.1 (0.5)	37.7 (0.4)	37.5 (0.3)
**Autoradiography (n)**	4	5	4	4	5	5	5	2
*Injected dose (MBq)*	4.7 (0.3)	5.9 (0.3)	7.9 (0.2)	8.0 (0.3)	6.3 (0.8)	5.7 (1.3)	7.2 (0.6)	5.9 (1.7)
*Weight (g)*	23.1 (2.1)	24.9 (2.6)	26.6 (2.7)	28.5 (4.2)	24.7 (3.4)	23.0 (1.2)	25.7 (2.5)	26.7 (3.5)
*Glucose (mmol/l)*	11.0 (2.3)	10.4 (0.9)	10.3 (1.8)	11.2 (1.9)	10.0 (1.1)	9.5 (2.5)	7.8 (1.3)	7.5 (1.9)
*Temperature (*°*C)*	37.9 (0.2)	35.2 (2.5)	37.8 (0.2)	36.6 (1.8)	35.4 (2.2)	36.0 (1.8)	37.7 (0.2)	37.6 (0.1)
**Immunohistochemistry**								
*6E10 (n)*	4	5	4	4	5	5	5	2
*Iba-1 (n)*	4	5	4	4	5	5	5	2

Data are presented as arithmetic means (standard deviations). TG = transgenic; WT = wild-type.

### Study deviations

During the study, one 6-month-old APP_Swe_-PS1_dE9_ WT mouse and one 17-month-old Tg2576 TG mouse died during the PET scans. In addition, SUV and SUV_Glu_ were not calculated for two Tg2576 mice due to missing weight or blood glucose measurements.

### Cerebral glucose metabolism measured by [^18^F]FDG PET

**Figure 2:**
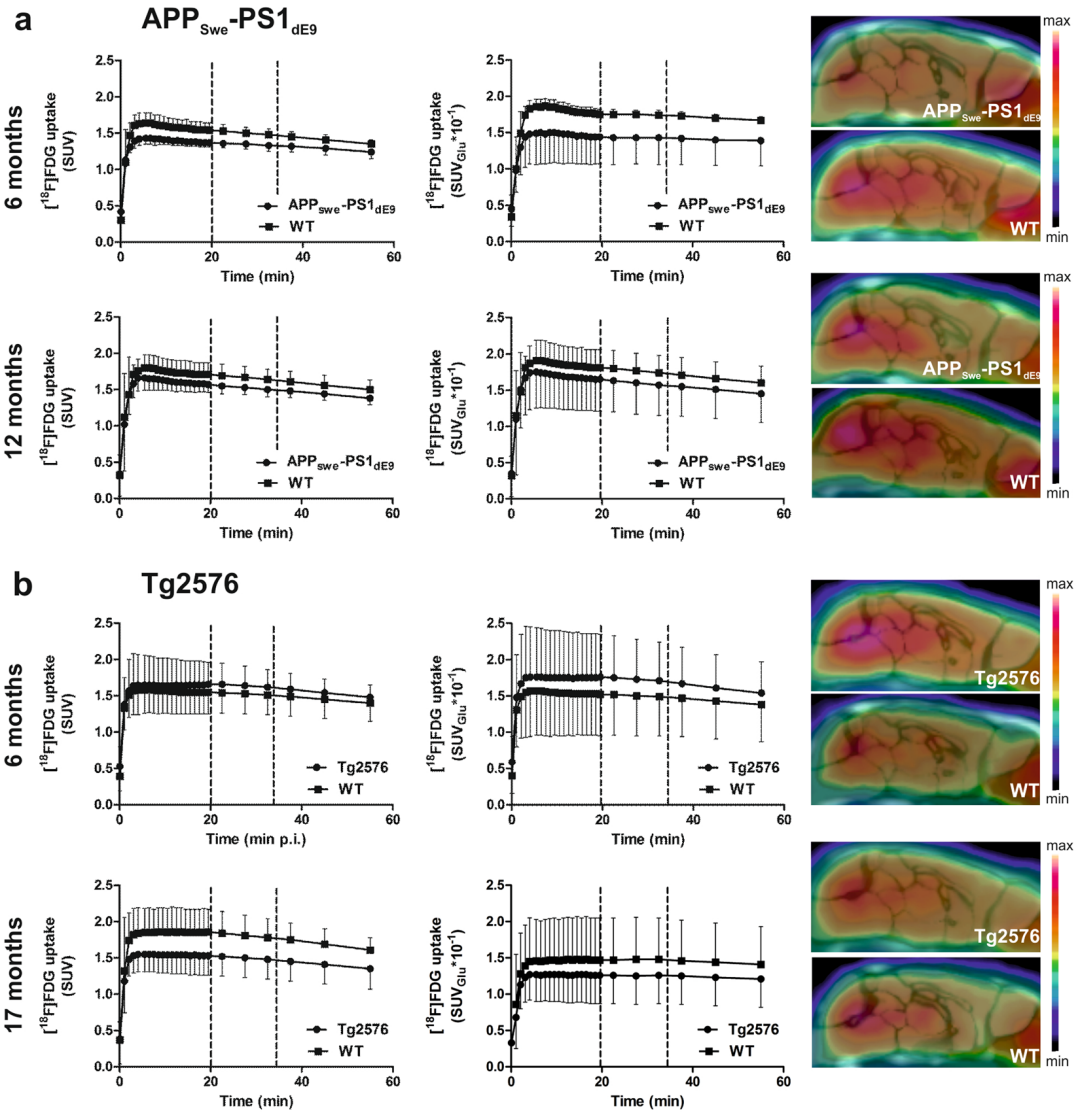
Mean time-activity curves and summed (20–35 min post-injection) PET images of cerebral [^18^F]FDG uptake in (**a**) APP_Swe_-PS1_dE9_ mice and wild-type (WT) controls at 6 and 12 months, and (**b**) Tg2576 mice and WT controls at 6 and 17 months. [^18^F]FDG was quantitated as standardized uptake values (SUVs) and SUVs corrected for individual blood glucose levels (SUV_Glu_). The dashed line represents the time frame used for summed PET images (20–35 min post-injection).

Brain [^18^F]FDG uptake was rapid, exhibiting a similar pattern in all groups, with the highest [^18^F]FDG accumulation in the midbrain region. In addition, high extracranial uptake was observed rostral to the brain in the Harderian glands (Fig. 2).

#### APP_Swe_-PS1_dE9_ mice

**Figure 3:**
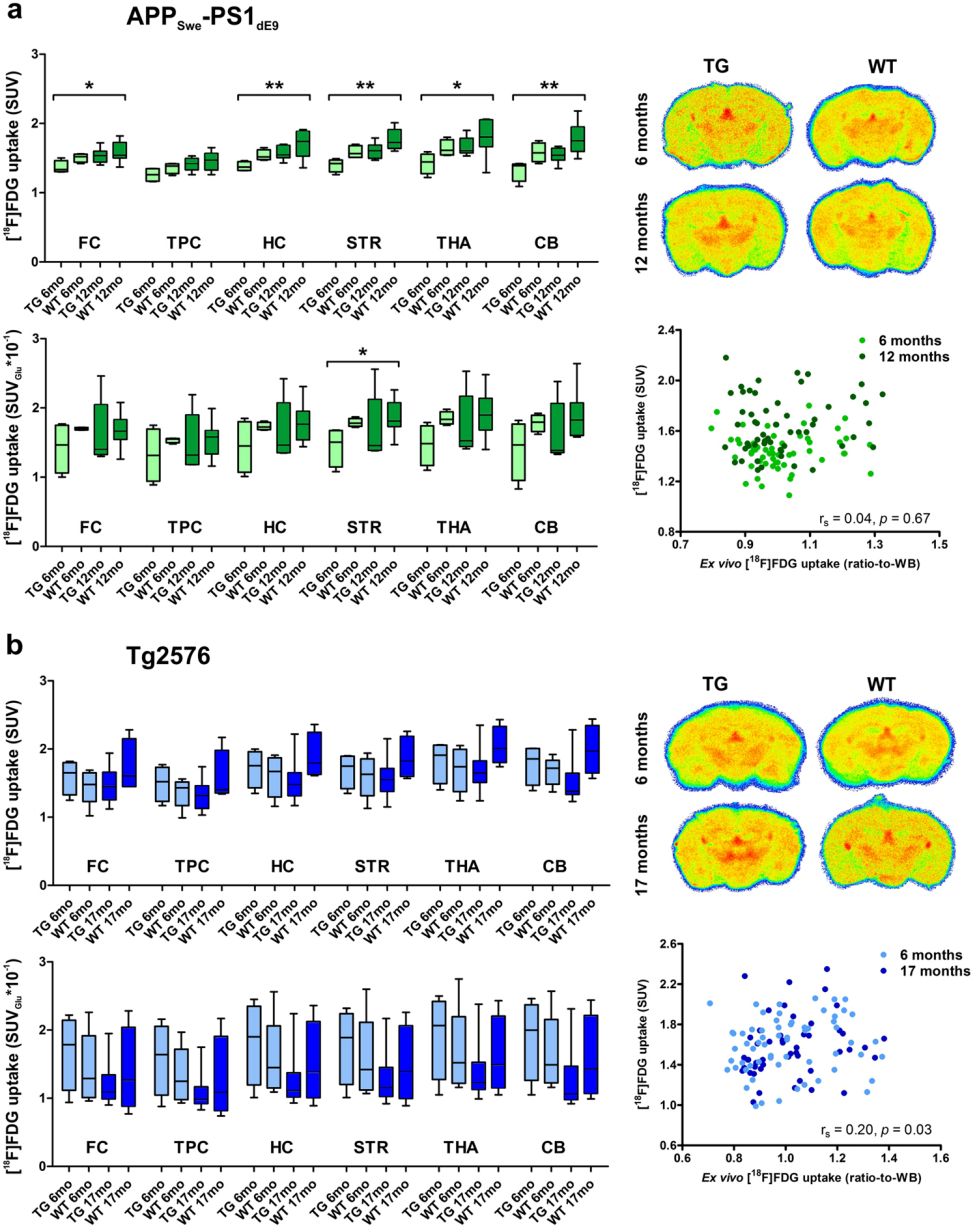
Regional *in vivo* [^18^F]FDG uptake as standardized uptake values (SUVs) and SUVs corrected for individual blood glucose levels (SUV_Glu_), representative *ex vivo* [^18^F]FDG autoradiographs, and scatter plots presenting the correlation of *in vivo* and *ex vivo* [^18^F]FDG uptake from all analysed regions quantitated as SUV-ratios to whole brain (SUVR_WB_) for (**a**) APP_Swe_-PS1_dE9_ and (**b**) Tg2576 transgenic (TG) mice and wild-type (WT) controls. Data are expressed as medians, upper and lower quartiles, minimums, and maximums for each group. Differences between the four groups (i.e. TG 6 months, WT 6 months, TG 12/17 months and WT 12/17 months) were tested using the non-parametric Kruskal-Wallis test (*p < 0.05; **p < 0.01). ARG = autoradiography; CB = cerebellum; FC = frontal cortex; HC = hippocampus; STR = striatum; THA = thalamus; TPC = tempo-parietal cortex.

APP_Swe_-PS1_dE9_ mice had lower median SUVs and SUV_Glu_s at both time points compared to age-matched WT mice (Fig. 3a; Table 2). Significant differences in regional [^18^F]FDG uptake (as SUVs) were detected at the level of whole brain (WB), frontal cortex (FC), hippocampus (HC), striatum (STR), thalamus (THA), and cerebellum (CB). When SUVs were normalized for individual glucose levels, variation within genotypes and age groups increased, and significant difference was found only in the STR. However, after subsequent comparison of the pre-defined pairs with multiple comparison test, significant differences were not found regardless of quantification method.

**Table 2:** [^18^F]FDG uptake in transgenic (TG) APP_Swe_-PS1_dE9_ and wild-type (WT) mice semi-quantitated as standardized uptake values (SUVs) and SUVs corrected for individual blood glucose levels (SUV_Glu_).

SUV	6 months		12 months		p	
	TG (n = 4)	WT (n = 4)	TG (n = 8)	WT (n = 10)		
WB	1.33 (1.30–1.42)	1.48 (1.44–1.59)	1.50 (1.43–1.64)	1.67 (1.52–1.85)	*0*.*027*	*
FC	1.33 (1.30–1.46)	1.52 (1.44–1.56)	1.53 (1.44–1.60)	1.54 (1.50–1.72)	*0*.*047*	*
TPC	1.26 (1.16–1.35)	1.38 (1.28–1.42)	1.42 (1.33–1.49)	1.47 (1.33–1.57)	*0*.*091*	ns
STR	1.42 (1.30–1.47)	1.56 (1.51–1.68)	1.60 (1.50–1.68)	1.73 (1.64–1.91)	*0*.*004*	**
HC	1.37 (1.32–1.45)	1.51 (1.48–1.62)	1.55 (1.50–1.68)	1.74 (1.53–1.88)	*0*.*009*	**
THA	1.45 (1.28–1.55)	1.61 (1.54–1.76)	1.60 (1.57–1.78)	1.80 (1.66–2.05)	*0*.*020*	*
CB	1.39 (1.16–1.42)	1.58 (1.45–1.72)	1.54 (1.47–1.64)	1.75 (1.59–1.95)	*0*.*004*	**
**SUV**_**Glu**_ *10 ^**−1**^	**TG (n = 4)**	**WT (n = 4)**	**TG (n = 8)**	**WT (n = 10)**	**p**	
WB	1.44 (1.05–1.73)	1.69 (1.65–1.76)	1.38 (1.33–2.02)	1.72 (1.51–1.92)	*0*.*17*	ns
FC	1.46 (1.06–1.75)	1.70 (1.68–1.72)	1.40 (1.32–2.05)	1.66 (1.54–1.86)	*0*.*44*	ns
TPC	1.31 (0.95–1.69)	1.55 (1.49–1.56)	1.32 (1.18–1.90)	1.58 (1.33–1.67)	*0*.*52*	ns
STR	1.50 (1.14–1.68)	1.78 (1.73–1.85)	1.46 (1.35–2.07)	1.81 (1.73–2.08)	*0*.*03*	*
HC	1.45 (1.07–1.80)	1.72 (1.69–1.79)	1.46 (1.35–2.07)	1.77 (1.54–1.95)	*0*.*24*	ns
THA	1.48 (1.17–1.74)	1.83 (1.77–1.95)	1.53 (1.45–2.17)	1.89 (1.68–2.14)	*0*.*12*	ns
CB	1.46 (1.06–1.75)	1.80 (1.66–1.89)	1.38 (1.35–2.06)	1.82 (1.60–2.07)	*0*.*11*	ns

Data are presented as medians and interquartile ranges. Differences between the four groups (TG, WT, 6 months and 12 months) were tested using the non-parametric Kruskal-Wallis test (*p < 0.05; **p < 0.01). CB = cerebellum; FC = frontal cortex; HC = hippocampus; ns = non-significant; STR = striatum; THA = thalamus; TPC = tempo-parietal cortex; WB = whole brain.

Significant differences in regional *ex vivo* [^18^F]FDG uptake ratios-to-WB were detected between groups only at the level of the parietal cortex (PC) and HC (see Supplementary Table S1). After comparing the pre-defined pairs with multiple comparison test, significance was still observed in the HC between APP_Swe_-PS1_dE9_ and WT mice at 6 months (p < 0.05).

#### Tg2576 mice

Only small differences between Tg2576 and WT mice, and high variation within all groups were detected in Tg2576 mice at 6 and 17 months, and no significant differences were observed in any of the analysed brain regions (Fig. 3b; Table 3). Similar to APP_Swe_-PS1_dE9_ mice, correcting SUVs for individual blood glucose levels further increased intragroup variation.

**Table 3:** [^18^F]FDG uptake in transgenic (TG) Tg2576 and wild-type (WT) mice semi-quantified as standardized uptake values (SUVs) and SUVs corrected for individual blood glucose levels (SUV_Glu_).

SUV	6 months		17 months		p	
	TG (n = 4^#^)	WT (n = 5)	TG (n = 9)	WT (n = 4)		
Brain	1.71 (1.36–1.85)	1.56 (1.26–1.78)	1.47 (1.30–1.61)	1.76 (1.57–1.94)	*0*.*23*	ns
FC	1.65 (1.33–1.80)	1.48 (1.23–1.65)	1.45 (1.25–1.66)	1.60 (1.45–2.15)	*0*.*46*	ns
TPC	1.52 (1.23–1.73)	1.43 (1.17–1.51)	1.32 (1.13–1.46)	1.40 (1.35–1.98)	*0*.*58*	ns
STR	1.75 (1.42–1.90)	1.63 (1.32–1.85)	1.55 (1.38–1.72)	1.82 (1.60–2.19)	*0*.*40*	ns
HC	1.75 (1.43–1.96)	1.67 (1.29–1.87)	1.48 (1.32–1.65)	1.79 (1.63–2.24)	*0*.*26*	ns
THA	1.91 (1.49–2.05)	1.74 (1.37–1.99)	1.65 (1.51–1.83)	2.01 (1.80–2.33)	*0*.*26*	ns
CB	1.85 (1.47–2.01)	1.72 (1.49–1.84)	1.38 (1.33–1.65)	1.97 (1.64–2.35)	*0*.*10*	ns
**SUV**_**Glu**_ *10 ^**−1**^	**TG (n = 4** ^**#**^ **)**	**WT (n = 5)**	**TG (n = 8** ^**¤**^ **)**	**WT (n = 4)**	**p**	
Brain	1.84 (1.16–2.19)	1.35 (1.10–1.97)	1.09 (1.00–1.37)	1.35 (0.98–1.98)	*0*.*46*	ns
FC	1.79 (1.12–2.14)	1.29 (1.01–1.91)	1.10 (0.99–1.34)	1.27 (0.88–2.04)	*0*.*64*	ns
TPC	1.64 (1.04–2.06)	1.25 (0.98–1.72)	0.99 (0.92–1.17)	1.09 (0.82–1.91)	*0*.*48*	ns
STR	1.89 (1.20–2.24)	1.42 (1.12–2.12)	1.16 (1.03–1.45)	1.39 (1.00–2.06)	*0*.*48*	ns
HC	1.90 (1.20–2.35)	1.45 (1.15–2.06)	1.11 (1.01–1.37)	1.39 (1.01–2.13)	*0*.*33*	ns
THA	2.07 (1.28–2.42)	1.52 (1.22–2.20)	1.23 (1.13–1.53)	1.49 (1.15–2.20)	*0*.*50*	ns
CB	2.00 (1.26–2.37)	1.49 (1.22–2.16)	1.07 (0.98–1.47)	1.43 (1.07–2.22)	*0*.*24*	ns

Data are presented as medians and interquartile ranges. Differences between the four groups (TG, WT, 6 months and 17 months) were tested using the non-parametric Kruskal-Wallis test. ^#^SUV was not calculated for one animal due to missing weight; ^¤^SUV_Glu_ was not calculated for one animal due to missing glucose measurement. CB = cerebellum; FC = frontal cortex; HC = hippocampus; ns = non-significant; STR = striatum; THA = thalamus; TPC = tempo-parietal cortex; WB = whole brain.

*Ex vivo* studies showed no significant differences in [^18^F]FDG uptake ratios-to-WB estimate in any brain region of Tg2576 mice between the groups (see Supplementary Table S1).

#### Correlation between SUV and SUV_Glu_

Correlation between regional [^18^F]FDG SUV and SUV_Glu_ values was also examined. For both APP_Swe_-PS1_dE9_ and Tg2576 models, a significant positive correlation was found both for pooled regional data (see Supplementary Fig. S1), and when brain regions were analysed separately (See Supplementary Table S2).

### *Ex vivo* [^18^F]FDG uptake

For APP_Swe_-PS1_dE9_, no significant differences in peripheral ^18^F-radioactivity uptake were found between TG and WT mice at any age (see Supplemental Fig. S2). In Tg2576 mice, significant differences in [^18^F]FDG uptake (as SUVs) were detected between age groups and genotypes in brown adipose tissue (BAT) (p = 0.04), and (as SUV_Glu_) in the brain (p = 0.04); however, after comparing the pre-defined pairs with multiple comparison test, no significant differences were observed anymore.

### Beta-amyloid deposition

#### APP_Swe_-PS1_dE9_ mice

**Figure 4:**
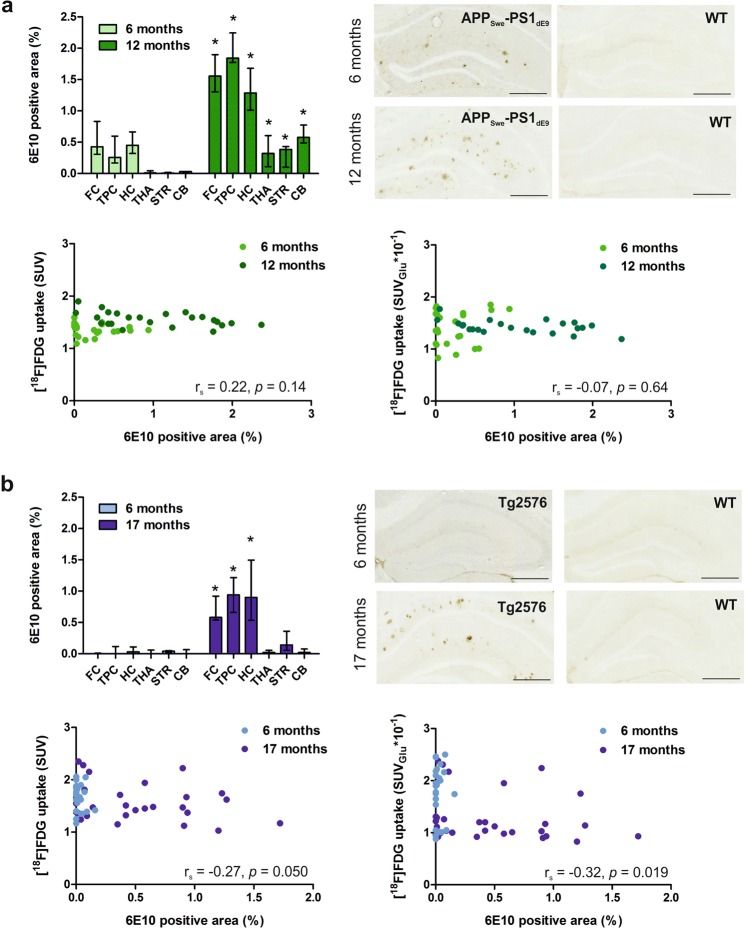
Cerebral beta-amyloid deposition evaluated as the regional 6E10-positive area (%), representative 6E10-stained hippocampal sections of transgenic (TG) and wild-type (WT) mice, and scatterplots presenting the correlation between regional beta-amyloid deposition and regional [^18^F]FDG uptake in (**a**) APP_Swe_-PS1_dE9_ mice at 6 and 12 months and (**b**) Tg2576 mice at 6 and 17 months. Data are presented as medians and interquartile ranges. Differences between TG 6 months and TG 12/17 months were tested using the non-parametric Mann-Whitney U-test (*p < 0.05). Scale bar = 500 µm.CB = cerebellum; FC = frontal cortex; HC = hippocampus; STR = striatum; THA = thalamus; TPC = tempo-parietal cortex.

APP_Swe_-PS1_dE9_ mice exhibited abundant Aβ deposition in all evaluated brain regions at 6 months, and a significant increase was seen in all regions at 12 months (Fig. 4a; p < 0.05). A weak positive correlation was found between the regional 6E10-positive area and [^18^F]FDG SUV uptake when pooled data from all brain regions and time points were examined (*n* = 48; r_S_ = 0.22, p = 0.14). When 6 months (*n* = 24; r_S_ = −0.33, p = 0.11) and 12 months (*n* = 24; r_S_ = −0.44, p = 0.028) were analysed separately, a moderate significant negative correlation was found at 12 months. For SUV_Glu_, no correlation was found between the variables for pooled data (*n* = 48; r_S_ = −0.07, p = 0.64) or at 6 months (*n* = 24; r_S_ = 0.05, p = 0.83), whereas a weak negative correlation was present at 12 months (*n* = 24; r_S_ = −0.34, p = 0.11).

#### Tg2576 mice

In Tg2576 mice, only sparse Aβ deposition around the brain was observed at 6 months, however, deposition increased significantly in the FC, TPC, and HC at 17 months (Fig. 4b, p < 0.05). Even at 17 months, deposition was approximately 2- to 3-fold lower in the cortical regions than in APP_Swe_-PS1_dE9_ mice at 12 months. When data from all evaluated brain regions and time points were pooled (*n* = 54), Tg2576 mice exhibited a weak negative correlation between 6E10-positive amyloid load and [^18^F]FDG uptake for both SUVs (*n* = 54; r_S_ = −0.27, p = 0.050) and SUV_Glu_ (*n* = 54; r_S_ = −0.32, p = 0.019). Similar weak correlations were gained when 17-month-old Tg2576 mice were analysed separately (*n* = 30; SUV: r_S_ = −0.20, p = 0.29; SUV_Glu_: r_S_ = −0.25, p = 0.18).

### Reactive microgliosis

#### APP_Swe_-PS1_dE9_ mice

**Figure 5:**
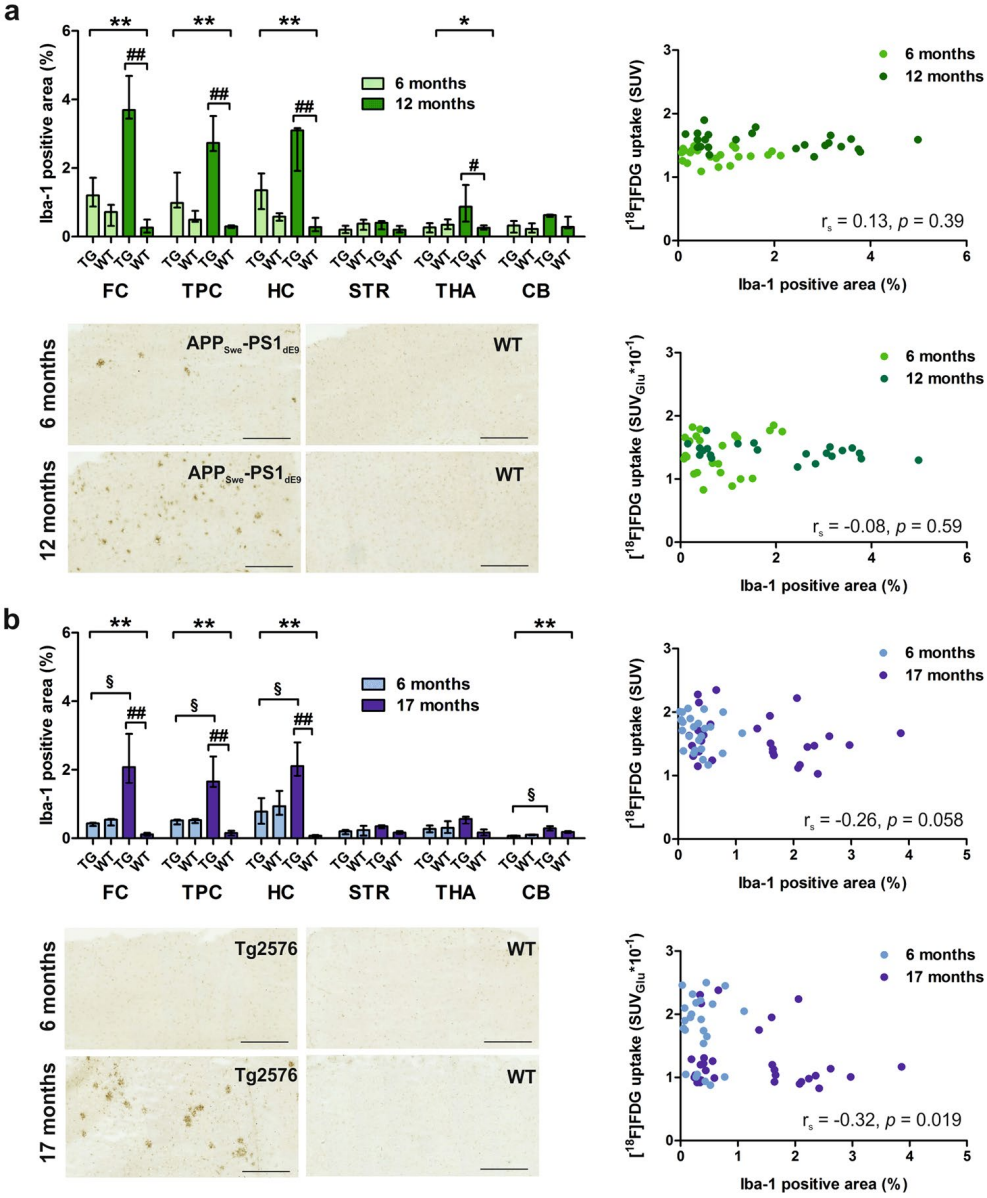
Cortical microgliosis evaluated as the Iba-1-positive area (%), representative anti-Iba-1-stained cortical sections of transgenic (TG) and wild-type (WT) mice, and scatterplots presenting the correlation between the regional Iba-1-positive area and regional [^18^F]FDG uptake in (**a**) APP_Swe_-PS1_dE9_ mice at 6 and 12 months and (**b**) Tg2576 mice at 6 and 17 months. Data are presented as medians and interquartile ranges. CB = cerebellum; FC = frontal cortex; HC = hippocampus; STR = striatum; THA = thalamus; TPC = tempo-parietal cortex. Differences between the four groups (i.e. TG 6 months, WT 6 months, TG 12/17 months and WT 12/17 months) were tested using the non-parametric Kruskal-Wallis test (*p < 0.05; **p < 0.01). When significant difference was found, Dunn’s multiple comparison test was used to compare four ad hoc pairs for between group differences: ^#^p < 0.05; ^##^p < 0.01 (TG 12/17 months vs. WT 12/17 months); ^§^p < 0.05 (TG 6 months vs. TG 17 months). Scale bar = 500 µm.

In APP_Swe_-PS1_dE9_ mice_,_ the Iba-1-positive area was larger than in WT mice already at 6 months, increased further at 12 months (Fig. 5a), and had a strong positive correlation with Aβ pathology at both time points (see Supplementary Fig. S3). The observed differences were statistically significant in the FC, TPC, HC, and THA. After comparing the pre-defined pairs with multiple comparison test, a significant difference (p < 0.05) was found between 12-month-old TG and WT mice in the FC, TPC, HC, and THA (Marked with # in Fig. 5a).

Similar to 6E10, no correlation was found between the Iba-1-positive area and [^18^F]FDG SUV uptake for APP_Swe_-PS1_dE9_ mice using pooled data from all regions and time points (*n* = 48; r_S_ = 0.13, p = 0.39) or at 6 months (*n* = 24; r_S_ = −0.13, p = 0.55). However, a weak negative correlation was found at 12 months (*n* = 24; r_S_ = −0.31, p = 0.15). For SUV_Glu_, no correlation was found between the variables for the pooled data (*n* = 48; r_S_ = −0.08, p = 0.59) or 6-month age group (*n* = 24; r_S_ = 0.07, p = 0.76), but a weak negative correlation was present at 12 months (*n* = 24; r_S_ = −0.32, p = 0.13).

#### Tg2576 mice

In Tg2576 mice, the regional anti-Iba-1-positive area was comparable to WT mice at 6 months, but increased significantly at 17 months (Fig. 5b). The observed differences were statistically significant in the FC, TPC, HC, and CB. After comparing the pre-defined pairs with multiple comparison test, significant (p < 0.05) differences were seen between 6- and 17-month-old TG mice in the FC, PTC, HC, and CB (marked with § in Fig. 5b), and between 17-month-old TG and WT mice in the FC, PTC, and HC (Marked with # in Fig. 5b). Iba-1-positive area had a strong positive correlation with Aβ pathology at 17 months (r_s_ = 0.75, p < 0.0001), but no correlation was observed at 6 months (r_s_ = 0.18, p = 0.34; see Supplementary Fig. S2).

Using pooled data, Tg2576 mice had a weak negative correlation between Iba-1-positive microgliosis and [^18^F]FDG uptake (*n* = 54; SUV: r_S_ = −0.26, p = 0.058; SUV_Glu_: r_S_ = −0.32, p = 0.019). Weak correlations were also found when the 6-month (*n* = 24; SUV: r_S_ = −0.33, p = 0.11; SUV_Glu_: r_s_ = −0.12, p = 0.58) and 17-month age groups (*n* = 30; SUV: r_S_ = −0.13, p = 0.50; SUV_Glu_: r_S_ = −0.19, p = 0.32) were analysed separately.

## Discussion

A full back-translation from clinical to preclinical studies would require clinical AD-related hypometabolism to be expressed in a mouse model of AD chosen for drug development. In our study, APP_Swe_-PS1_dE9_ mice had lower median [^18^F]FDG uptake in all brain regions compared to age-matched WT mice at 6 and 12 months, whereas no such differences between genotypes were seen in Tg2576. In addition to the APP_Swe_-PS1_dE9_ model in this study, decreased regional [^18^F]FDG uptake has previously been reported for APP-PS1-21 mice in both a cross-sectional^16^ and our previous longitudinal study at 12 months^6^. In contrast, previous studies using the same APP_Swe_-PS1_dE9_ model reported either unaltered glucose uptake at 9-month-old TG mice^22^ or increased regional [^18^F]FDG uptake in 2- to 4-month-old mice before the onset of amyloid deposition^12^. This finding of early hypermetabolism is in line with increased [^18^F]FDG uptake in amyloid-negative subjects with mild cognitive impairment^23,24^, suggesting the presence of a compensatory mechanism at the time of initial neuronal dysfunction in the early phase of AD. In our study, the first time-point for APP_Swe_-PS1_dE9_ mice was 6 months, when abundant amyloid deposition was already present. Taken together, these two studies suggest that [^18^F]FDG PET could detect similar temporal alterations in cerebral glucose metabolism in the APP_Swe_-PS1_dE9_ model as what are present in early and late phases of clinical AD, however, differences are modest and not correlated with amyloid pathology.

Neuroinflammation has been suggested to be one factor behind the observed increase in [^18^F]FDG uptake in mice, as activated glia cells also utilize [^18^F]FDG^25,26^. However, when we investigated the relationship between reactive microgliosis and [^18^F]FDG uptake using pooled data from both time points, there was no or weak negative correlation in TG mice. A similar weak negative relationship was reported previously for APP-PS1-21 mice^27^.

Our results showed an additional trend towards increased regional [^18^F]FDG uptake in both APP_Swe_-PS1_dE9_ and WT mice from 6 to 12 months of age. This finding is not in line with the cerebral hypometabolism reported during aging in humans^28^. In aging TG mice, this temporal increase could be explained by an elevation of Aβ pathology and accompanying microgliosis, as a significant increase in 6E10 reactivity and Iba-1-positive microgliosis was observed in multiple brain regions from 6 to 12 months. However, as a similar temporal increase was present in WT mice, and no positive correlation was found between [^18^F]FDG SUVs and Iba-1 or 6E10 staining in TG mice, these changes were most likely caused by general physiological alterations related to murine aging rather than the expression of transgenes. Similar findings in WT mice have also been reported in previous imaging studies^6,11,25,27^, in which the increased metabolic activity was speculated to be associated with age-related neuroinflammation and enlarged microglial soma accompanied by increased translocator protein accumulation during normal aging in rodents.

In the present study, the Tg2576 model did not exhibit significant differences in [^18^F]FDG uptake and demonstrated higher intragroup variability than expected based on the previous studies. Previous studies with this strain have had varying results. Unchanged cerebral [^18^F]FDG uptake was reported at 13–15 months^18^, and increased tracer uptake was demonstrated in 7-month-old Tg2576 mice compared to age-matched WT controls^9^. Furthermore, [^18^F]FDG uptake was decreased, though not significantly, in 18-month-old Tg2576 mice **s**howing decreased trend in the [^18^F]FDG retention after SUVs were corrected with individual blood glucose values^29^. Based on this study and the conflicting results presented above, the Tg2576 model seems to be less attractive for future interventional studies than the APP_Swe_-PS1_dE9_ model.

Compared to many other imaging targets, [^18^F]FDG imaging in small animals offers additional challenges due to the quantification method and vast number of variables that affect animal physiology and, thus, [^18^F]FDG uptake^26^. Fasting of animals prior to [^18^F]FDG imaging reduces endogenous glucose levels, decreases tracer uptake in cardiac and skeletal muscle, and increases uptake in the brain^30^. For this study, 3 hours was chosen for fasting to avoid negative effects caused by overnight fasting, such as hormonal disturbances and substantial weight loss^31^. In addition, because mice consume two-thirds of their daily food intake during the night, we wanted to minimize the stress on the animals by starting the fasting in the morning^31^. The value of *in vivo* imaging for drug development lies in the possibility for longitudinal studies in which the effect of drug compounds can be evaluated in an animal model of the disease. As these include multiple imaging sessions and imaging of old and fragile TG animals, we did not consider prolonged fasting times feasible.

Quantification of [^18^F]FDG uptake in mice has been shown to be problematic. As absolute quantification and required blood sampling is rarely applicable, various semi-quantitative approaches, such as relative measures or SUVs, are commonly used. However, with these methods, the study protocol and environmental factors need to be standardized to ensure unbiased study outcomes. It has been recently suggested that normalizing SUVs with blood glucose levels would enable more reliable semi-quantification of [^18^F]FDG uptake, especially if the duration of fasting varies^26^. However, in our study, the duration of the fasting period was exactly the same in all animals, and correcting regional SUVs with the individual blood glucose values led to increased variability within all groups in both models. This might originate from the fact that our fasting time was not appropriate to stabilize the blood glucose levels in these models, and blood samples were taken before injection rather than during the uptake phase. However, even after 24-h fasting, a previous study failed to demonstrate significant decreases in cerebral glucose metabolism old Tg2576 animals^29^. In our experience, if careful standardization of environmental factors is performed and glucose levels do not significantly differ between the study groups, normalizing to blood glucose may not bring additional benefits.

Normalizing the regional uptake to a reference region void of pathology is also a widely used approach in [^18^F]FDG studies. However, because our 6E10 immunohistochemistry experiments revealed Aβ deposition in the CB of both investigated AD models, as well as differences in the CB SUVs detected in APP_Swe_-PS1_dE9_ mice at both ages, cerebellar reference region approach was not justified, and whole brain estimates were further calculated for the autoradiographs. Unfortunately, a standard ^18^F-activity curve allowing for better quantification, was not done during the data collection phase.

An obvious limitation of the study was a lack of longitudinal study design due to animal availability. In addition, an earlier time point for APP_Swe_-PS1_dE9_ mice would have given more information on the early pathological phase prior to Aβ deposition. To date, transgenic rat models of AD are also available, and due to their larger brain they provide more accurate PET images. However, as commercial mouse models are still more widely used, this study concentrated only on comparing commercially available mouse models. Different fasting times could have been tested for both models to clarify the tolerance for food deprivation, and adding blood glucose measurements to the baseline before fasting and closer to the time of [^18^F]FDG injection would have given more information about the effect of fasting and anaesthesia among the used mouse models. Immunohistochemical staining with neuronal and synaptic markers could have provided additional information about the observed [^18^F]FDG uptake.

In this study, by using the same experimental protocols and analysis methods, we were able to demonstrate that APP_Swe_-PS1_dE9_ and Tg2576 differ in regards to both abundance of Aβ pathology and neuroinflammation, and the observed changes in glucose metabolism at investigated time-points. Based on our findings, the APP_Swe_-PS1_dE9_ model could be more suitable for future longitudinal [^18^F]FDG imaging studies evaluating interventions targeting e.g. synaptic and neuronal restoration. However, considering the modest intergroup differences and difficulties reliably quantifying [^18^F]FDG uptake in mouse brain, the potential of more specific PET tracers, such as the novel tracers targeting synaptic vesicle glycoprotein 2 A^32^, should be investigated for evaluating neuronal dysfunction in AD models.

## Materials and Methods

### [^18^F]FDG synthesis

[^18^F]FDG (37 batches) was synthesized at the Radiopharmaceutical Chemistry Laboratory of Turku PET Centre using the FASTlab synthesizer (GE Healthcare, Waukesha, WI, USA) as described previously^6^. Radiochemical purity exceeded 95% in all syntheses.

### Experimental animals

The study was approved by the Regional State Administrative Agency for Southern Finland (ESAVI-2010-04454/ym-23). Animal care complied with the principles of the International Council of Laboratory Animal Science (ICLAS) and reported in compliance with the ARRIVE guidelines. Females from two different TG mouse models, APP_Swe_-PS1_dE9_ (B6.Cg-Tg(APP_Swe_, PSEN1_dE9_)85Dbo/Mmjax, The Jackson Lab) and Tg2576 (SJL-Tg(APPSWE)2576Kha, Taconic Europe), were used in this study. Animals were treated as described in Supplementary materials (See Supplementary Methods) and allocated to groups by genotype and availability as illustrated in Fig. 1a.

### PET imaging

The design of the PET study is presented in Fig. 1b using black and red text. [^18^F]FDG PET scans at predefined time points of 6 months (both models), 12 months (APP_Swe_-PS1_dE9_), and 17 months (Tg2576) were performed with an Inveon Multimodality PET/computed tomography (CT) scanner (Siemens Medical Solutions USA, Knoxville, TN) for isoflurane-anaesthetized mice. All PET scans were performed during the light phase between 9 a.m. and 1 p.m., and environmental conditions were standardized throughout the study as illustrated in Fig. 1b. CT scan was first acquired for attenuation correction, then, dynamic PET data were collected for 60 min in 3D list mode with an energy window of 350–650 keV. The data were then reconstructed using Fourier rebinning and a two-dimensional filtered back-projection reconstruction algorithm, and divided into 51 time frames (30 × 10 s, 15 × 60 s, 4 × 300 s, and 2 × 600 s) and decay corrected to the time of injection.

### PET image analysis

Volume of interest (VOI) based analysis was performed using Inveon Research Workplace Image Analysis software v. 4.1 and 4.2 (Siemens Medical Solutions USA, Knoxville, TM.), and a predefined VOI library was manually drawn to a mouse brain MRI template^33^. PET images were first co-registered with CT images and MRI template using rigid registration. After alignment, predefined VOIs for the FC, TPC, HC, CB, THA, STR, and WB were loaded onto the co-registered MRI template. Imaging data were semi-quantified as standardized uptake values (SUVs) by dividing the regional activity by the ratio of injected dose and body weight of the mouse. Time activity curves were acquired for each VOI, and mean SUVs were calculated for a time period of 20–35 min post-injection. Based on recent suggestions^26^, SUVs were also corrected for individual blood glucose levels to obtain SUV_Glu_ values (SUV_Glu_ = SUV × individual blood glucose value) for better semi-quantification of [^18^F]FDG uptake in the absence of a suitable reference region.

### *Ex vivo* tissue counting and autoradiography

The experimental conditions for *ex vivo* studies were identical to those of *in vivo* imaging as illustrated in Fig. 1b with black and blue text. The experiments were performed 7 ± 1 days after the PET scans for animals from the second batch (indicated with blue color in Fig. 1a). To reach an equivalent time point used in the PET analysis, [^18^F]FDG was allowed to accumulate for 30 min before animals were sacrificed as described previously^6^. The brain, heart, liver, muscle, and brown fat were dissected, weighed, and measured for ^18^F-radioactivity in a NaI(Tl) well counter (3 × 3 inches, Bicron, Newbury, OH, USA). Measured radioactivity was corrected for decay and background and expressed as SUV and SUV_Glu_.

After measuring, the brain was frozen in chilled isopentane and cut into 20-μm coronal sections using a cryomicrotome (Microm HM 500 OM, Walldorf, Germany). Approximately 10 glass slides with a minimum of eight sections per slide from four levels were cut from each animal, exposed to an imaging plate and scanned with the Fuji Analyzer BAS5000 (resolution 25 μm). Regions of interest (ROIs) were drawn to the FC, PC, TC, HC, STR, THA, and CB in the digitalized images on a minimum of eight sections/region for each animal. Because cerebellar reference region approach was not justified based on the cerebellar uptake and Immunohistochemical analysis, a mean count density for all of the analysed ROIs was calculated and used as an estimate of the WB uptake. Finally, the radioactivity uptake in each brain region was evaluated as a ratio-to-WB. All analyses were performed with a computerized image analysis program (Aida 4.22, Raytest Isotopenmessgeräte, GmbH, Straubenhardt, Germany).

### Immunohistochemical staining

From the second batch of mice (indicated with blue color in Fig. 1a), cryosections were stored at −20 °C for subsequent immunohistochemical staining with monoclonal antibody against Aβ_1–16_ (6E10) and ionized calcium-binding adapter molecule 1 (anti-Iba-1) expressed in reactive microglia. Detailed quantification methods, protocols, used reagents and antibodies are described in Supplementary materials (see Supplementary Methods and Supplementary Table S3).

### Data analysis and statistical procedures

Descriptive statistics were expressed as arithmetic means with standard deviation (SD) for each group in Table 1. Due to the relatively small sample size and the distribution of the numeric data, the results were described using medians and interquartile ranges and analysed using non-parametric methods. Initial differences in regional [^18^F]FDG uptake and anti-Iba-1 staining were tested between both genotypes and time points (i.e. between 4 groups) using the Kruskal-Wallis test. If significant difference was found, Dunn’s multiple comparison test was used to compare differences between four ad hoc pairs (i.e. TG 6 months vs WT 6 months; TG 6months vs TG 12/17 months; TG 6 months vs WT 12/17 months; WT 6 months vs WT 12/17 months). As no Aβ deposits were present in WT brain, only differences between TG mice at different ages were tested using the non-parametric Mann-Whitney U-test. Associations between regional [^18^F]FDG uptake and Aβ deposition or Iba-1-positive reactive gliosis were analysed using Spearman’s correlation. The distribution of data and all statistical analyses were performed in GraphPad Prism v. 5.01 (GraphPad Software, v. 5.01, San Diego, CA, USA). Differences were considered significant when p < 0.05.

## Supplementary information

Electronic Supplementary Material

## Data Availability

The datasets generated during and/or analysed during the study are available from the corresponding author on reasonable request.
