# Supplementary material for: Effect of genotype and age on cerebral [18F]FDG uptake varies between transgenic APPSwe-PS1dE9 and Tg2576 mouse models of Alzheimer’s disease
Source: Sci Rep. 2019 Apr 5;9:5700. doi: 10.1038/s41598-019-42074-4 (PMC6450965; doi:10.1038/s41598-019-42074-4)
Supplement: Supplementary file 1 — Electronic Supplementary Material [file 41598_2019_42074_MOESM1_ESM.docx]

**ELECTRONIC SUPPLEMENTARY MATERIAL**

**Effect of genotype and age on cerebral [^18^F]FDG uptake varies between transgenic
APP_Swe_-PS1_dE9_ and Tg2576 mouse models of Alzheimer’s disease**

**Journal: Scientific Reports**

Anniina Snellman*^1,2^, Jatta S. Takkinen^1,2,3^, Francisco R. Lopez-Picon^1,2^, Olli Eskola^4^, Olof Solin^4,5,6^, Juha O. Rinne^7,8^, and Merja Haaparanta-Solin^1,2^

^1^MediCity Research Laboratory, University of Turku, Tykistökatu 6 A, FI-20520 Turku, Finland

^2^PET Preclinical Laboratory, Turku PET Centre, University of Turku, Tykistökatu 6 A, FI-20520 Turku, Finland

^3^Doctoral Programme in Clinical Research, University of Turku, Turku, Finland

^4^Radiopharmaceutical Chemistry Laboratory, Turku PET Centre, University of Turku, Kiinamyllynkatu 4-8, FI-20520 Turku, Finland

^5^Accelerator Laboratory, Turku PET Centre, Åbo Akademi University, Kiinamyllynkatu 4-8, FI-20520 Turku, Finland

^6^Department of Chemistry, University of Turku, Vatselankatu 2, FI-20500 Turku, Finland

^7^Turku PET Centre, Turku University Hospital, Kiinamyllynkatu 4-8, FI-20520 Turku, Finland

^8^Division of Clinical Neurosciences, Turku University Hospital, Kiinamyllynkatu 4-8, FI-20520 Turku, Finland

***Corresponding author:** Anniina Snellman, PhD, Tykistökatu 6A, FI-20520 Turku, Finland, Tel: + 358 2 3337020, Fax: +358 29 450 5040, E-mail: [aepakk@utu.fi](mailto:aepakk@utu.fi)

**SUPPLEMENTARY METHODS**

**Animal models**

Two transgenic animal mouse models, Tg2576 and APP_Swe_-PS1_dE9_, were used in this study. Briefly, Tg2576 mice carry the 695-isoform of the amyloid precursor protein (APP) gene with Swedish double mutation (APP_Swe_, K670N/M671L) and start to exhibit Aβ deposition and associated reactive gliosis by 11-13 months of age, whereas the APP_Swe_-PS1_dE9_ double TG strain expresses both APP_Swe_ and presenilin-1 with deletion of exon 9, resulting in earlier initiation of Aβ deposition typically at 6 months of age. All animals were housed at 21 ± 3°C in 55 ± 15% humidity with a light period from 6:00 a.m. to 6:00 p.m and free access to tap water and soya-free chow (RM3 (E) soya, 801710, Special Diets Service, Essex, UK). Mice were weighed before each experimental session and body temperature and blood glucose levels (Glu) measured before each PET scan using a microprobe rectal thermometer (Physitemp Instruments, Inc., USA) and Accu-Chek Aviva Nano (Roche Diagnostics, USA), respectively. All animals were fasted for 3 hours, taken to the laboratory 45 minutes and anesthesized 30 minutes prior to [^18^F]FDG injection.

**Quantification of beta-amyloid deposition and microgliosis**

A minimum of one slide containing 8-10 sections was stained for each mouse. Stained sections were scanned using the 3DHISTECH Slide Scanner 250 and evaluated by CaseViewer software version 1.3 (3DHISTECH, Budapest, Hungary). Six different brain regions (FC, TPC, HC, THA, STR, and CB) were analysed from each mouse to estimate the amount of regional Aβ and microgliosis. From each mouse, 2−3 sections (1−4 snapshots each) were captured for each region at 15x magnification using CaseViewer software. The snapshots were converted into 8-bit images and thresholded individually by comparing them to the original images. Finally, the percentage of 6E10 or anti-Iba-1-positive area out of the total image area was calculated using Image J (Wayne Rasband, National Institute of Health, MD, USA), and mean values for each region were used for correlation analysis.

**SUPPLEMENTARY FIGURES**

**
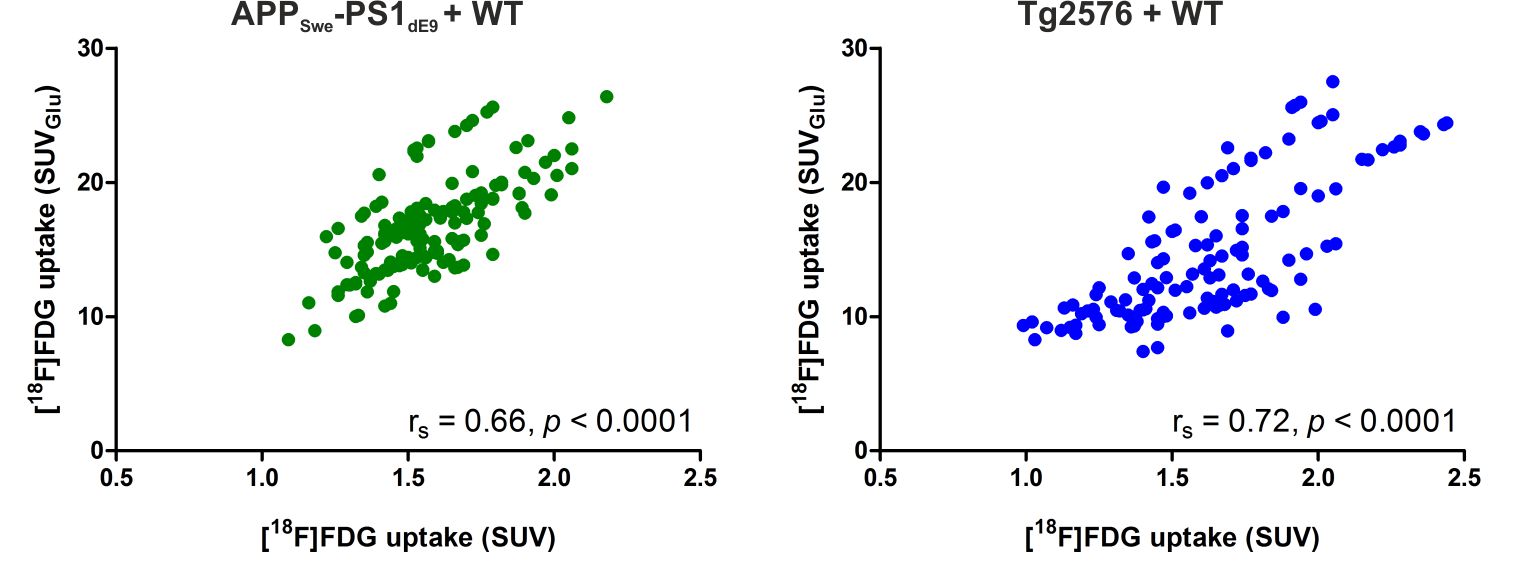
**

**Supplementary Figure S1**

Correlation between obtained standardized uptake values (SUV) and SUVs normalized for individual blood sugar levels (SUV_Glu_) in APP_Swe_-PS1_dE9_ and wild-type (WT) mice, and Tg2576 and WT mice. Significant positive correlation between SUV and SUV_Glu_ was found for both APP_Swe_-PS1_dE9_ (r_s_ = 66, p < 0.0001) and Tg2576 (r_s_ = 0.72, p < 0.0001) models. For this figure, SUV and SUV_Glu_ values from both transgenic and WT mice, and data from all analysed brain regions was pooled for clarity (correlations calculated individually for each analysed brain region are reported in detail in Supplementary Table S1).

**
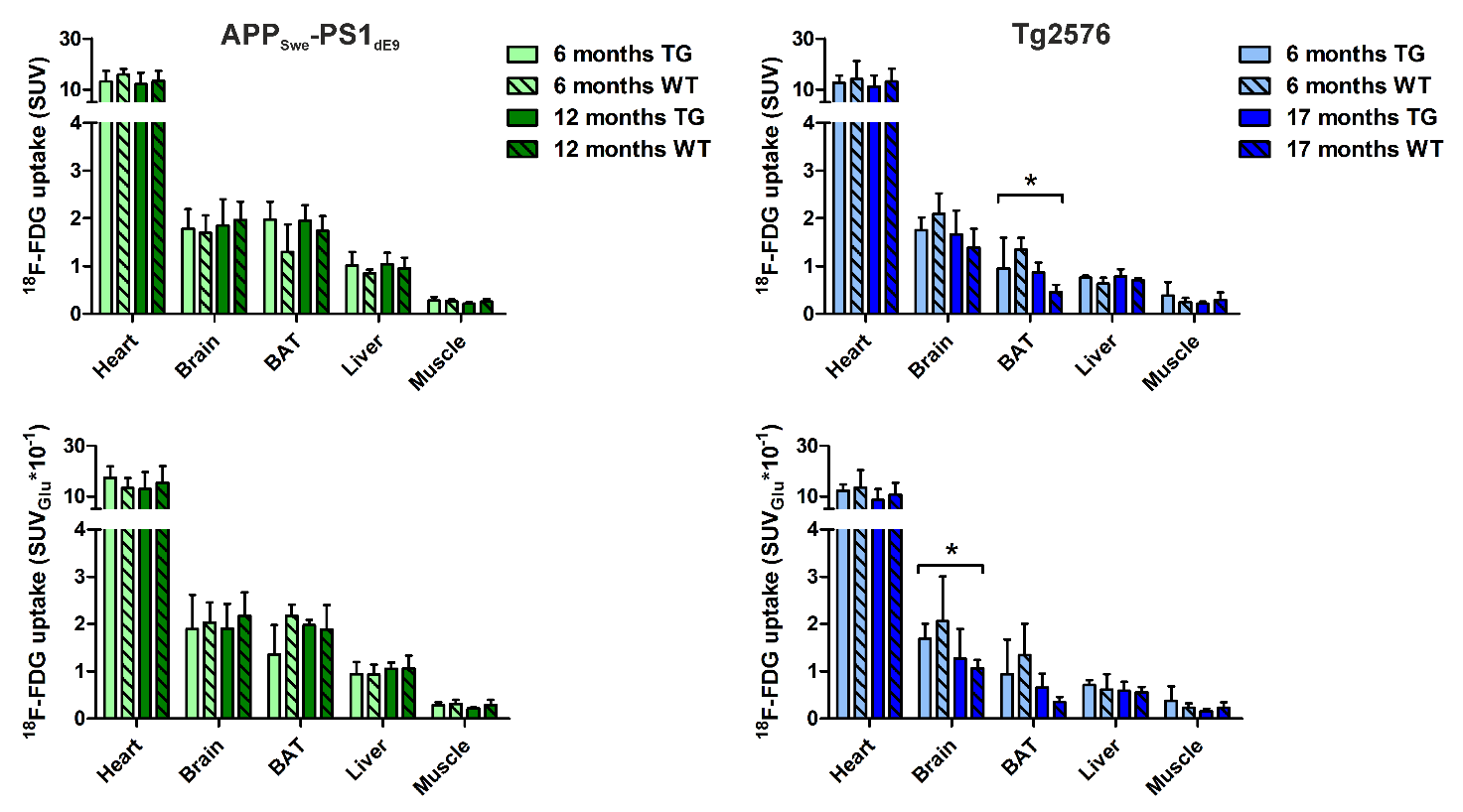
**

**Supplementary Figure S2**

*Ex vivo* tissue counting results from transgenic (TG) APP_Swe_-PS1_dE9_, Tg2576, and wild type (WT) mice quantified as standardized uptake values (SUV) or SUVs corrected for individual blood glucose levels (SUV_Glu_). No statistically significant differences were detected in brain, heart, brown adipose tissue (BAT), liver and muscle between the evaluated age groups of 6 and 12 months or genotypes in APP_Swe_-PS1_dE9_ and WT mice. In Tg2576 and WT mice, [^18^F]FDG uptake in BAT as SUV, and brain as SUV_Glu_ showed significant difference between groups (*p* = 0.04), however, significance was lost after correcting for multiple comparisons.


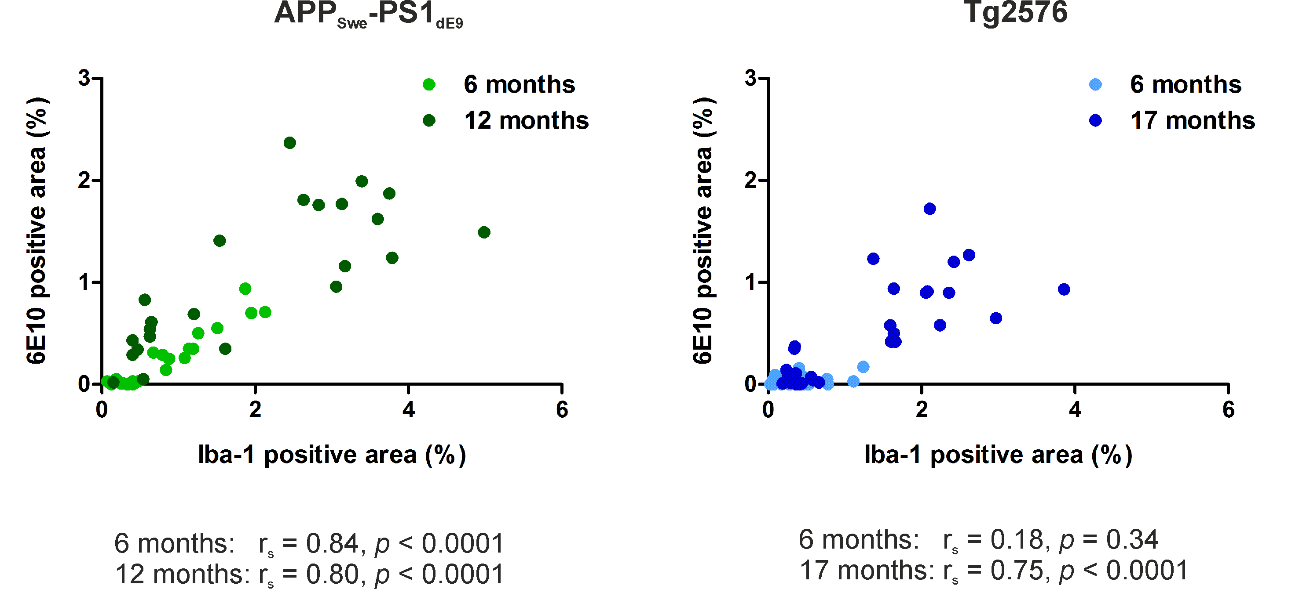


**Supplementary Figure S3**

Correlation between 6E10 positive beta-amyloid deposits and Iba-1 positive microgliosis in APP_Swe_-PS1_dE9_ and Tg2576 transgenic mice. In APP_Swe_-PS1_dE9_ mice, strong positive correlation between regional beta-amyloid deposition and microgliosis was detected both at 6 months (r_s_ = 0.84, p < 0.0001) and 12 months of age (r_s_ = 0.80, p < 0.0001). In Tg2576 mice, no correlation was observed yet at 6 months (r_s_ = 0.18, p = 0.34), whereas strong correlation (r_s_ = 0.75, p < 0.0001) was present at 17 months.

**SUPPLEMENTARY TABLES**

**Supplementary Table S1**

Regional *ex vivo* digital autoradiography results presented as [^18^F]FDG binding ratios (R), using whole brain (WB) estimate as a reference region. Data are presented as medians and interquartile ranges. Differences between groups are tested with non-parametric Kruskal-Wallis test (*p < 0.05), and if significance was found additional Dunn’s test for multiple comparisons was performed (#p < 0.05 after multiple comparisons). Abbreviations: CB = cerebellum; FC = frontal cortex; HC = hippocampus; ns = non-significant; R_WB_ = binding ratio-to-whole brain; STR = striatum; TG = transgenic; THA = thalamus; TPC = tempo-parietal cortex; WB = whole brain; WT = wild type.

|  | **APP_Swe_-PS1_dE9_** | | | | |  |  |
| --- | --- | --- | --- | --- | --- | --- | --- |
|  | **6 months** | |  | **12 months** | |  |  |
| **R_WB_** | **TG** | **WT** |  | **TG** | **WT** | **p** |  |
| FC | 1.00 (0.94–1.01) | 0.98 (0.94–1.07) |  | 1.02 (1.01–1.04) | 0.98 (0.95–1.02) | 0.34 | ns |
| PC | 0.96 (0.91–0.99) | 1.04 (0.97–1.05) |  | 1.01 (1.01–1.05) | 0.94 (0.91–1.00) | 0.04 | * |
| TC | 0.94 (0.92–0.95) | 0.96 (0.91–0.99) |  | 0.98 (0.97–1.00) | 0.97 (0.96–1.01) | 0.08 | ns |
| STR | 1.25 (1.21–1.29) | 1.19 (1.12–1.21) |  | 1.20 (1.11–1.30) | 1.26 (1.17–1.31) | 0.28 | ns |
| HC | 0.99 (0.98–1.02)^#^ | 0.89 (0.88–0.92)^#^ |  | 0.94 (0.91–0.97) | 0.92 (0.87–0.95) | 0.02 | * |
| THA | 1.06 (0.96–1.10) | 1.09 (1.07–1.18) |  | 1.09 (1.07–1.6) | 1.09 (1.07.1.19) | 0.61 | ns |
| CB | 0.94 (0.88–1.02) | 0.83 (0.80–0.95) |  | 0.87 (0.84–0.93) | 0.89 (0.86–0.94) | 0.31 | ns |
|  |  |  |  |  |  |  |  |
|  | **Tg2576** | | | | |  |  |
|  | **6 months** | |  | **17 months** | |  |  |
| **R_WB_** | **TG** | **WT** |  | **TG** | **WT** | **p** |  |
| FC | 0.87 (0.8-0.97) | 0.91 (0.82–0.99) |  | 0.89 (0.86–0.92) | 0.88 (0.85–0.91) | 1.00 | ns |
| PC | 0.89 (0.84–0.93) | 0.88 (0.79–0.92) |  | 0.86 (0.84–0.89) | 0.87 (0.83–0.91) | 0.94 | ns |
| TC | 0.86 (0.80–0.96) | 0.86 (0.79–0.92) |  | 0.89 (0.86–0.94) | 0.90 (0.85–0.94) | 0.85 | ns |
| STR | 1.22 (1.19–1.26) | 1.29 (1.20–1.33) |  | 1.22 (1.13–1.30) | 1.20 (1.09–1.31) | 0.47 | ns |
| HC | 0.98 (0.91–0.99) | 1.05 (0.94–1.14) |  | 1.01 (1.00–1.03) | 0.94 (0.89–0.98) | 0.32 | ns |
| THA | 1.09 (1.02–1.17) | 1.15 (1.10–1.19) |  | 1.10 (1.02–1.27) | 1.20 (1.20–1.21) | 0.11 | ns |
| CB | 0.86 (0.77–0.93) | 0.87 (0.80–0.92) |  | 0.88 (0.86–0.92) | 1.04 (1.03–1.04) | 0.13 | ns |

**Supplementary Table S2**

Correlation between regional standard uptake values without (SUV), and with normalization for blood glucose levels (SUV_Glu_) evaluated using Spearman’s correlation. Strong and significant positive correlation was found in all brain regions in both models. Abbreviations: CB = cerebellum; FC = frontal cortex; HC = hippocampus; STR = striatum; THA = thalamus; TPC = tempo-parietal cortex.

|  | **APP_swe_-PS1_dE9_** | |  | **Tg2576** | |
| --- | --- | --- | --- | --- | --- |
|  | **SUV vs. SUV_Glu_** | |  | **SUV vs. SUV_Glu_** | |
|  | **Spearman r** | ***p*** |  | **Spearman r** | ***p*** |
| FC | 0.60 | 0.0013 |  | 0.75 | 0.0001 |
| TPC | 0.70 | <.0001 |  | 0.68 | 0.0007 |
| HC | 0.57 | 0.0026 |  | 0.67 | 0.001 |
| THA | 0.65 | 0.0003 |  | 0.67 | 0.0009 |
| STR | 0.67 | 0.0002 |  | 0.68 | 0.0008 |
| CB | 0.62 | 0.0007 |  | 0.72 | 0.0002 |

**Supplementary Table S3**

Details of the used protocols for beta-amyloid and Iba-1 immunohistochemistry (IHC). PFA = paraformaldehyde; BSA = bovine serum albumin; NGS = normal goat serum; PBS = phosphate buffered saline

|  | **Beta-amyloid IHC** | **anti-Iba-1 IHC** |
| --- | --- | --- |
| **Sections** | Fresh frozen | Fresh frozen |
| **Post fixation** | 4% PFA, 30 min | 4% PFA, 30 min |
| **Peroxidase blocking** | 0.3% H_2_O_2_ in 10% MeOH, 20 min | 0.3% H_2_O_2_ in 10% MeOH, 30 min |
| **Antigen retrieval** | > 95% Formic acid, 10 min | NA |
| **Blocking** | 0.3% Triton X-100, 2% BSA, 2% NGS in PBS (pH 7.4), 60 min | 0.3% Triton X-100, 2% BSA, 2% NGS in PBS (pH 7.4), 120 min |
| **Primary antibody** | 6E10, anti-Abeta_1-16_ (SIG-39320) | Anti-Iba-1 (CAT# 019-19741) |
| **Manufacturer** | Covance Inc. | Wako Pure Chemical Industries, Ltd |
| **Dilution** | 1:500 | 1:1000 |
| **Incubation time** | O/N, + 8C | O/N, + 8C |
| **Wash** | PBSt, 3 x 5 min | PBSt, 3 x 5 min |
| **Secondary antibody** | Biotin-SP-conjugate AffiniPure Goat Anti-mouse IgG | Biotin-Goat-anti-rabbit IgG |
| **Manufacturer** | Jackson Immuno Research Laboratories | Invitrogen |
| **Dilution** | 1:500 | 1:500 |
| **Incubation time** | 30min, RT | 60 min, RT |
| **Wash** | PBSt, 3x10 min | PBSt, 3x10 min |
| **Avidin-Peroxidase conjugate** | 30 min | 60 min |
| **Substrate** | 3,3'-Diaminobenzidine | 3,3'-Diaminobenzidine |
